# The Influence of Ionizing Radiation on Itraconazole in the Solid State

**DOI:** 10.1208/s12249-014-0185-9

**Published:** 2014-08-27

**Authors:** Katarzyna Dettlaff, Przemysław Talik, Grzegorz Spólnik, Witold Danikiewicz, Magdalena Ogrodowczyk

**Affiliations:** 1 Department of Pharmaceutical Chemistry, Poznan University of Medical Sciences, Grunwaldzka 6, 60-780 Poznań, Poland; 2 Department of Inorganic and Analytical Chemistry, Jagiellonian University, Collegium Medicum, Medyczna 9, 30-688 Kraków, Poland; 3 Institute of Organic Chemistry, Polish Academy of Sciences, Kasprzaka 44/52, 01-224 Warszawa, Poland

**Keywords:** antifungal azole, DSC, itraconazole, product radiolysis, radiation sterilization

## Abstract

The aim of this study was to investigate the ionizing radiation effects, in the form of an electron beam, on itraconazole (ITR) in the solid phase. It was found that the ITR, under the influence of a standard 25 kGy dose of radiation used for the sterilization of drug substances, decomposed at 0.4%. Moreover, a gentle change of colour and a decrease in melting point does not exceed pharmacopoeial standards causing that ITR can be sterilized by radiation method. The use of high 400 kGy radiation doses resulted in a 6.5% decomposition of the ITR and eight radiodegradation products were found. However, with the exception of differential scanning calorimetry (DSC), the X-ray diffraction, Fourier transform infrared spectroscopy (FT-IR) and ultraviolet-visible (UV-vis) methods showed no changes in the form and the morphology of the crystals. The structures of all those compounds were investigated. It was confirmed that the ITR decomposition takes place by dehalogenation (one of Cl atom elimination), the oxidation in isobutyl residue (beside the triazole ring) and C-O bond rupture.

## INTRODUCTION

**Fig. 1:**
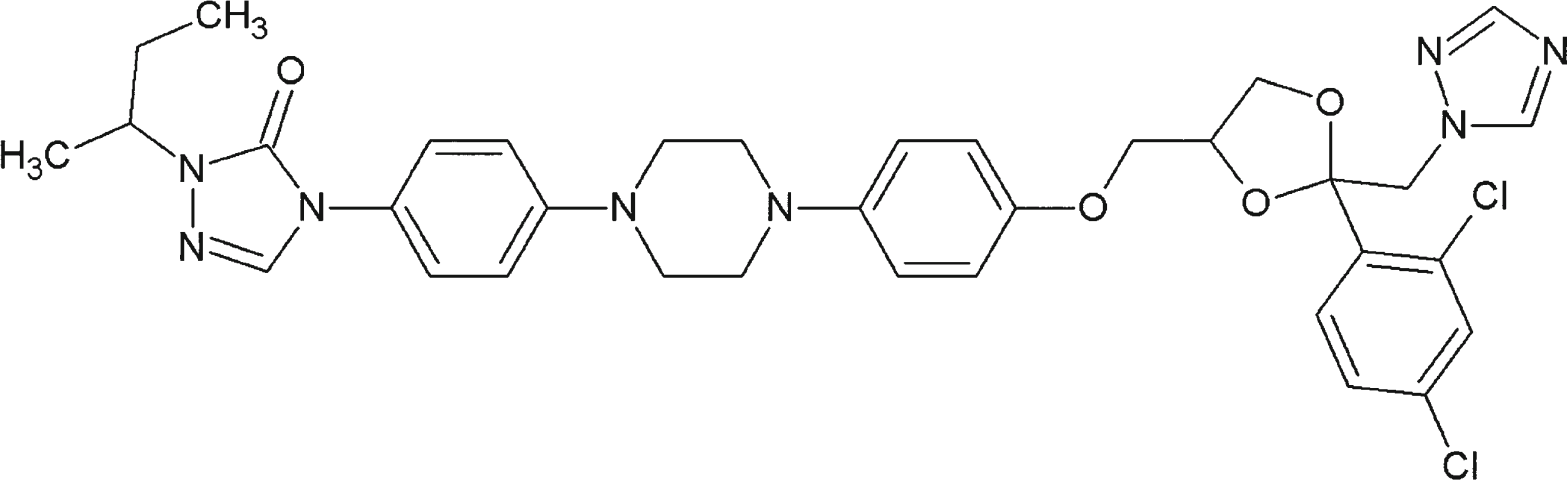
Structure of itraconazole (ITR)

Itraconazole (ITR) ((2*R*,4*S*)-1-(butan-2-yl)-4-{4-[4-(4-{[(2*R*,4*S*)-2-(2,4-dichloro phenyl)-2-(1*H*-1,2,4-triazol-1-ylmethyl)-1,3-dioxolan-4-yl]methoxy}phenyl)piperazin-1-yl] phenyl}-4,5-dihydro-1*H*-1,2,4-triazol-5-one) is a third-generation antifungal azole, a derivative of 1,2,4-triazole (Fig. 1). It is characterized by a broad spectrum of action and its activity was found against *Trichophyton* spp., *Microsporum* spp., *Epidermophyton floccosum*, *Candida* spp. (including *Candida albicans*, *Candida glabrata*, *Candida krusei*), *Malassezia* spp., *Cryptococcus neoformans*, *Histoplasma* spp., *Paracoccidioides brasiliensis*, *Blastomyces dermatitidis*, *Sporothrix schenckii*, *Aspergillus* spp., *Fonsecaea* spp., *Cladosporium* spp., *Geotrichum* spp., *Pseudallescheria boydii* and *Penicillium marnefei* (1–3). A usual dose between 100 and 400 mg daily is recommended orally (capsule) and parenterally (injections, solutions for infusion) to patients (4).

All parenteral drugs must be sterile. The radiation sterilization, which is one of the accepted methods of the European Pharmacopeia 6.0 (5), is applied more and more often (6,7).

Above all, it can be conducted at room or lower temperatures which allow for thermolabile drug sterilization. However, the most important limitation is the fact that the degradation of the drug molecule is possible. The factors causing the greatest damage during the irradiation process are radicals formed in the radiolysis reaction of water (*H*_2_ *O* ^· +^, ^·^ *OH*, *H*_3_ *O* ^+^, *H* ^·^, *O* ^·^, *H*_2_, *e* ^−^, *e*_*aq*_^−^) (6,8). Therefore, the irradiation of therapeutic substances should be carried out in the solid state (6,7,9). Although the first studies on the sterilization of drugs are dated back to the 70s of the twentieth century (they concerned most often thermolabile antibiotics of the penicillin group), many of them has not known radiation resistance yet. It is estimated that about 90% could be sterilized in the solid phase; however, it should be proved for each drug that ionizing radiation does not change any of its physicochemical properties (6). The mechanism of radiodegradation is often different than the previously known mechanism either of photo- or thermodegradation. An assumption that the resistance of ionizing radiation is similar in the group of drugs with similar structure cannot be made (6,9–13). The comprehensive assessment for X-ray methods, spectroscopy, thermal and chromatographic analysis only enables capturing all changes that may occur during the irradiation process of a therapeutic compound, in order to ensure the safety of therapy (6,9).

In the present paper, the effect of ionizing radiation as a beam of high energy electrons on the physicochemical properties of ITR in the solid phase has been investigated. A standard dose of radiation sterilization (25 kGy) and higher radiation doses (50–400 kGy) have been applied to understand the mechanism of ITR degradation and also to compare the results of previous radiochemical stability studies, involving four other derivatives of azole antifungals: clotrimazole, fluconazole, ketoconazole and miconazole nitrate (10–13).

## MATERIALS AND METHODS

### Materials

The ITR pure white standard was purchased from Shouguang Fukang Pharmaceutical Co., Ltd, China, assay 98.5–101.5% (in compliance with European Pharmacopeia 6.0—(5))—Fig. 1.

### Method of Exposure

Approximately 0.2 g of ITR was placed in a colourless glass vial of 5-mL capacity, closed with a plastic stopper and exposed to an e-beam from a linear electron accelerator, Elektronika 10/10 (electron beam 9.96 MeV; current intensity 6.2 μA), until doses of 25, 50, 100, 200 and 400 kGy were absorbed.

### Scanning Electron Microscopy

The scanning electron microscopy (SEM) analysis was made using a SEM 515 (Philips, The Netherlands) electron microscope with 14 mm working distance and 3–10 kV accelerating voltage. The ITR samples (about 2 mg) of 0 and 400 kGy were placed on specimen stubs and fixed with carbon tabs; then, they were sputter-coated with gold in a sputter coater, type SCD 050 Balzers. Selected pictures were processed by Digital Image Scanning System (DISS).

### Fourier Transform Infrared Spectroscopy

Two KBr discs were prepared by compressing the mixture of 1.00 mg of ITR (before and after irradiation—400 kGy) with 300 mg of KBr, in a Pye Unicam minipress. The spectra were recorded using the spectrometer IRAffinity-1 (Shimadzu, Japan) with KBr as a blank. The measurement parameters were as follows: range 500–4.000 cm^−1^, resolution 4.0 cm^−1^ and number of scans 40.

### Ultraviolet-Visible Spectrophotometry

The solutions were prepared by dissolving the ITR samples (from all irradiation doses 0–400 kGy) in methanol to a concentration of 0.02% *w*/*v*. The solutions were examined using a Perkin Elmer UV/VIS Lambda 20 spectrophotometer (SpectraLab Scientific Inc., Ontario, Canada), in 1-cm cells in the range 200–800 nm, using methanol as a blank.

### X-ray Diffractometry

The X-ray diffraction patterns for ITR powdered samples (0, 25 and 400 kGy) were performed on a Bruker AXS D8 Advance powder diffractometer with a Johansson focusing monochromator CuKα1 radiation (λ = 1.5406 Å) at 25°C for the angles from the range 5° ≤ 2θ ≤ 60° (step size 0.05°/1.0 s) and strip detector LynxEye, controlled by an IBM PC unit.

### Melting Point Determination

The melting points of ITR were determined by capillary method using MP 70 Melting Point System (Mettler Toledo). The measurements were performed for unirradiated and irradiated ITR samples (25, 50, 100, 200 and 400 kGy). Samples were heated from 25 to 175°C, in the range 155–175°C; heating rate was 1°C/min. The result was an average of three measurements.

### Differential Scanning Calorimetry

The differential scanning calorimetry (DSC) measurements were performed in a nitrogen atmosphere with a flow rate of 50 mL/min using an EXSTAR DSC 7020 apparatus (SII NanoTechnology Inc.) calibrated with indium and tin and equipped with DSC 7020 electric cooling unit. Samples of about 4.8 to 5.1 mg were precisely weighted in aluminium pans and sealed. The samples were first equilibrated at 30°C for 15 min, and thereafter, the melting behaviours were analyzed at a heating rate of 5°C/min. The measurements were performed at least three times and averaged.

### Ultra Performance Liquid Chromatography Coupled to High Resolution Mass Spectrometry

Ultra performance liquid chromatography coupled to >high resolution mass spectrometry (UPLC-HRMS) analysis was performed using the ultra-performance liquid chromatograph ACQUITY UPLC I-Class (Waters Inc.) coupled with a MALDISynapt G2-S HDMS (Waters Inc.) mass spectrometer equipped with an electrospray ion source and quadrupole-time-of-flight mass analyzer. UPLC separations was done with the 2.1 × 100 mm ACQUITY UPLC BEH C18 (1.7 μm) column (Waters Inc.). Solvent A was water and solvent B was acetonitrile. The gradient flow was used from the 30% of solvent B as the initial conditions to the 100% of solvent B at 13 min, and it was maintained for 1 min and then back to initial conditions. The flow rate was 0.3 mL/min. The samples (ITR 0, 25 and 400 kGy) were diluted in acetonitrile. The peak at 2.40 min is present in all chromatograms and corresponds to the solvent background.

The measurements were performed in positive ion mode with the sampling cone set to 40 V and capillary voltage set to 3,000 V. The MS^e^ experiments were set to collect the fragmentation spectra with the energy ramping from 30 to 50 eV.

## RESULTS AND DISCUSSION

**Fig. 2:**
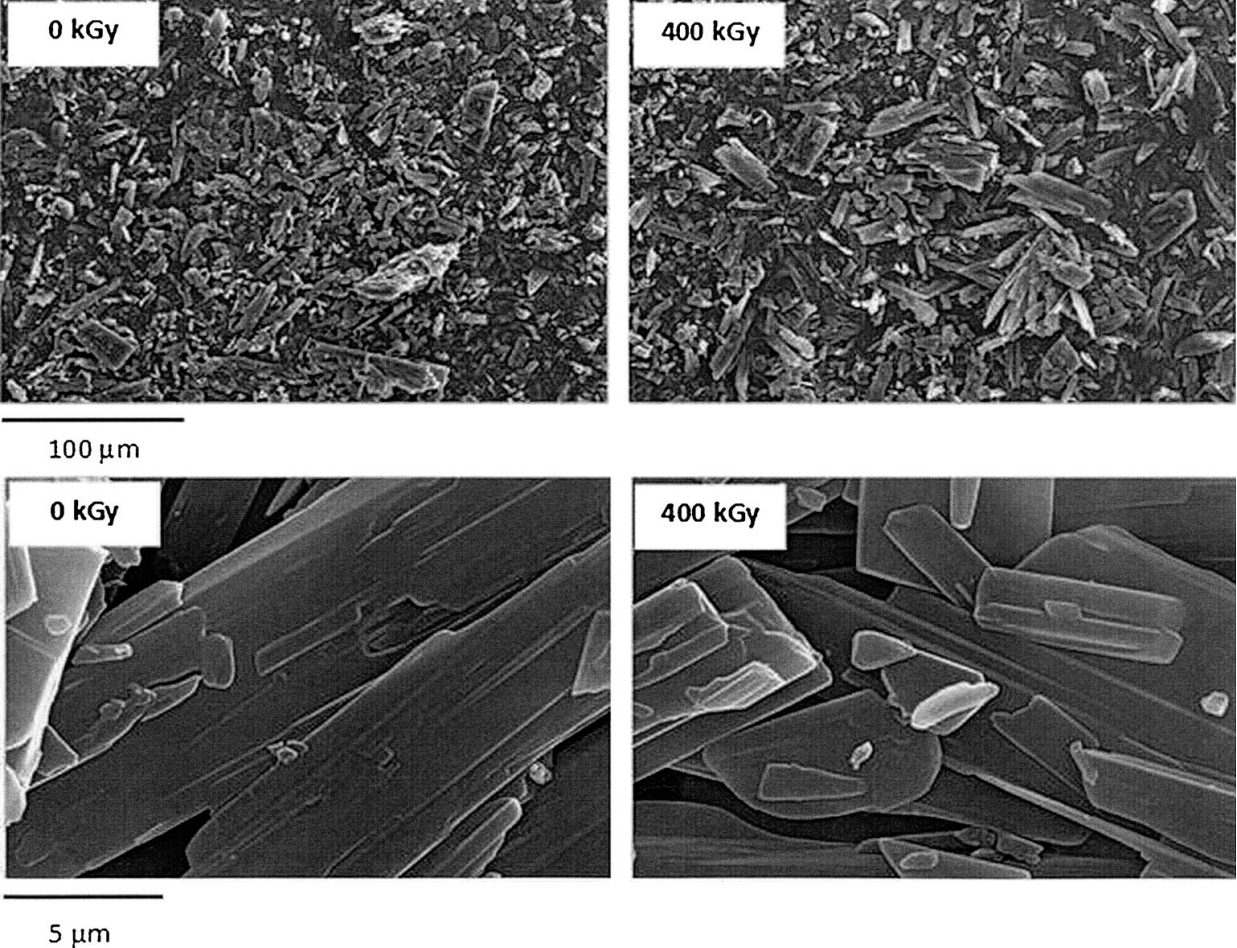
SEM photographs of ITR before (0 kGy) and after irradiation (400 kGy) in two magnifications

The first change of ITR colour from standard pure white to off-white and light grey was observed after irradiation with dose of 50 kGy and it was becoming more distinct and darker up to the grey-greenish colour (400 kGy) with the increase of radiation dose. To verify if there are changes in the dispersion degree or if there are a visible damages to the crystal morphology, the scanning electron microscope (SEM) was used. Our observations concerned two radiation doses 0 and 400 kGy. No such changes were observed (top images on Fig. 2) even in the larger magnification (bottom images).

### Spectroscopic Analysis

**Fig. 3:**
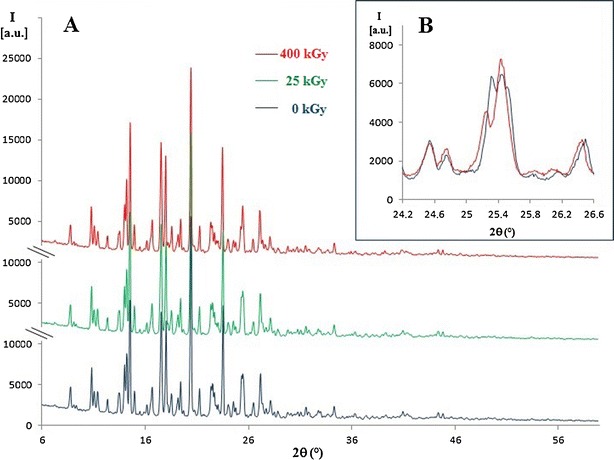
**a** X-ray diffractograms of ITR before (0 kGy) and after irradiation (doses 5 and 400 kGy), range 6.0–60.0°. **b** X-ray diffractograms of ITR before (0 kGy) and after irradiation (400 kGy), range 24.2–26.6°

To determine whether the colour of the compound is connected with the defects in the crystal lattice, the X-ray diffraction analysis of non-irradiated and irradiated (with doses of 25 and 400 kGy) ITR was performed. It was found that the location and intensity of the measured reflections were consistent (Fig. 3a). Only for sample exposed to dose 400 kGy slight changes in the range 25–26° can be observed (Fig. 3b).

**Fig. 4:**
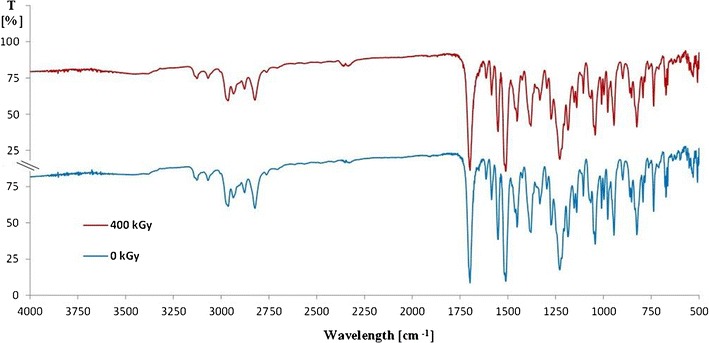
FT-IR spectra of ITR before (0 kGy) and after irradiation (400 kGy)

The next step was to analyze the spectroscopic studies. The Fourier transform infrared spectroscopy (FT-IR) spectra of ITR which were not subjected to ionizing radiation and which were irradiated with 400 kGy (that was 16 times higher than a standard dose) are consistent throughout the entire course and transmittance values—Fig. 4.

So either there were no changes in the ITR structure or the radiodegradation products were formed in such a small content. That is, they can be either compounds with very similar structures to ITR or they are its structural isomers. Taking into account the large mass of the molecule (705 Da), a small impurity content which differs from the parent compound with more or less a single bond (C-H or C-O) cannot be noticed in the mixture spectrum.

The spectrophotometric analysis of the ultraviolet and visible wavelengths allowed to conclude that the spectra of non-irradiated and irradiated ITR are consistent even at the highest dose. No new absorption band, as it was in the case of fluconazole (11) and clotrimazole (12), was observed. The absorbance decrease at the analytical wavelength for a 25-kGy dose was 0.32% and for a 400-kGy dose 1.08%. It also indicates the compound stability or formation of radiolysis products which contain the same chromophore groups as the parent compound.

#### Calorimetric Analysis

**Fig. 5:**
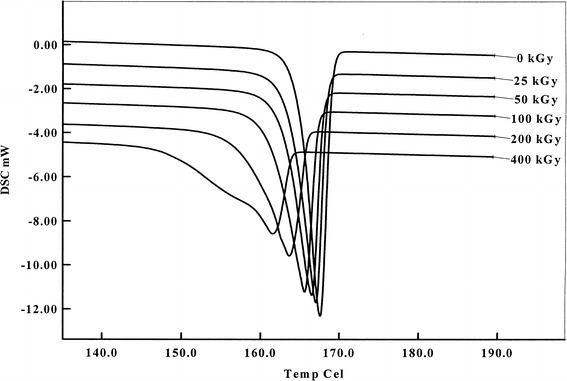
DSC curves of ITR before and after irradiation

**Fig. 6:**
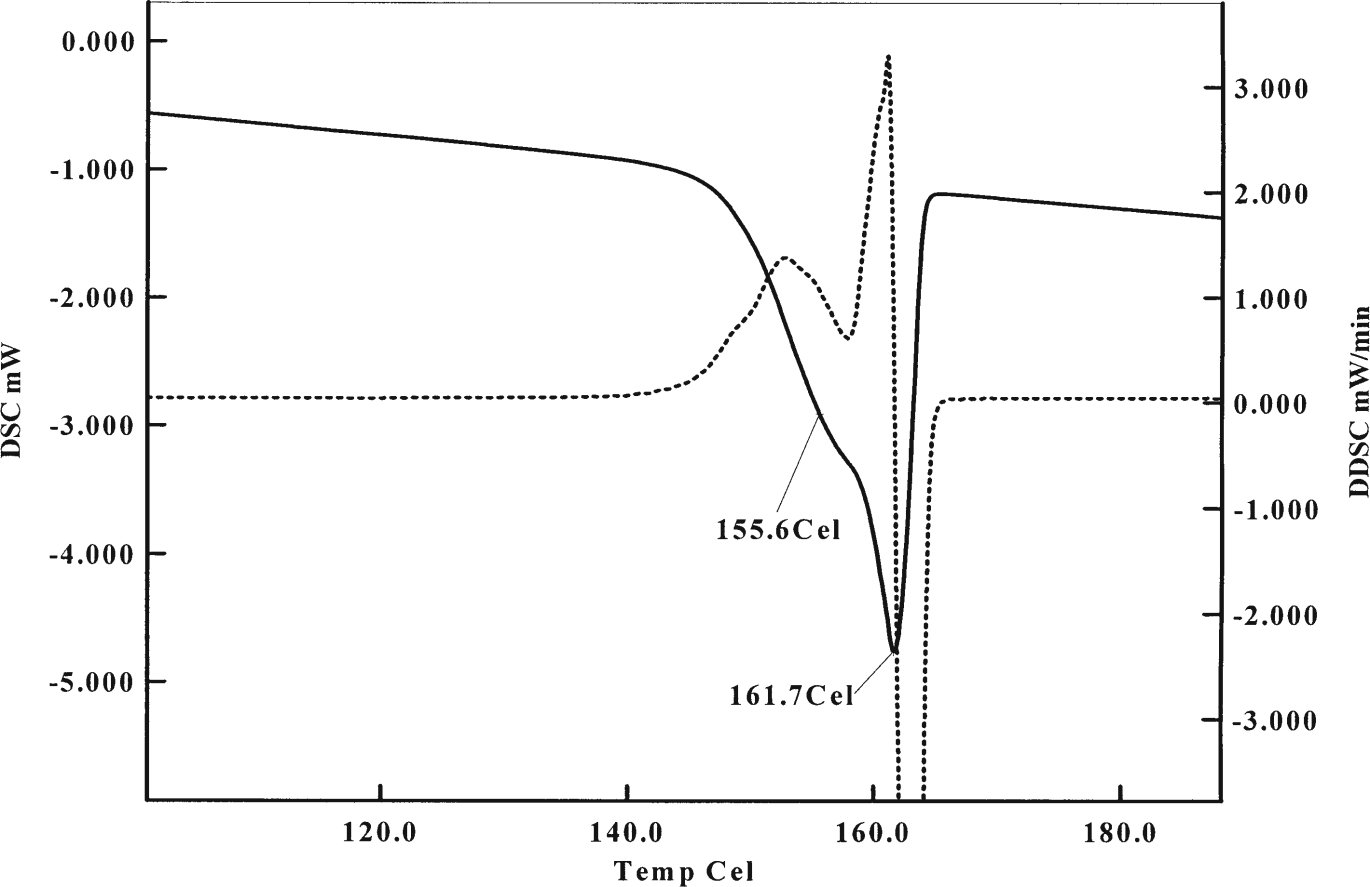
DSC curve of ITR irradiated with 400 kGy dose with additional peak at 155.6°C

Figure 5 shows the heating curves of ITR samples under study. The melting point represented by *T*_onset_ and *T*_max_ shifts to the lower temperature side as the radiation level is increased, reaching values, for minimum 0 kGy and maximum 400 kGy dose, 164.8 and 167.6°C and 155.7 and 161.6°C, respectively (Table I). It means that the sample composition has changed and consists of other compounds/impurities formed during the e-beam exposure. In consequence, the melting enthalpies Δ*H* decreased from −89.3 J/g (0 kGy) to −71.3 J/g (400 kGy). The presence of those radiodegradation products is also clearly visible in Fig. 6 where additional, overlapping peak, obtained from 400 kGy sample, can be seen at *T*_max_ = 155.6°C.

**Table I:** Thermal Analysis Data Obtained by Means of DSC and Capillary Method; the Capillary Technique was Performed in Accordance with the Requirements of European Pharmacopeia 6.0 (5). All Values are Averaged at Least from Three Measurements; the Values in Parentheses are Standard Deviation (SD)

Dose (kGy)	DSC			Capillary method	
	*T*_onset_ (°C)	T_max_ (°C)	Δ*H* (J g^−1^)	*T*_onset_ (°C)	*T*_endset_ (°C)
0	164.8 (0.40)	167.6 (0.00)	−89.3 (1.27)	167.3 (1)	169.7 (1)
25	163.5 (0.08)	167.1 (0.06)	−82.1 (0.81)	166.5 (1)	169.9 (1)
50	162.7 (0.31)	166.4 (0.13)	−81.2 (1.88)	166.0 (1)	168.5 (4)
100	161.1 (0.10)	165.5 (0.13)	−80.1 (1.82)	165.1 (1)	168.2 (4)
200	158.7 (0.50)	164.0 (0.23)	−79.3 (1.33)	162.3 (1)	166.0 (4)
400	155.7 (0.53)	161.6 (0.10)	−71.3 (0.61)	160.5 (2)	161.0 (7)
Difference^a,b^	9.1^a^	6.0^a^	20.16^b^	6.8^a^	8.7^a^
Norm (5)	–	–	–	166.0	170.0

^*a*^Δ*T*_onset/max/endset_ = *T*_onset/max/endset_^0kGy^ − *T*_onset/max/endset_^400kGy^

^*b*^(Δ*H*_0_− Δ*H*_400_) / Δ*H*_0_ (%)

#### Chromatographic Analysis

**Fig. 7:**
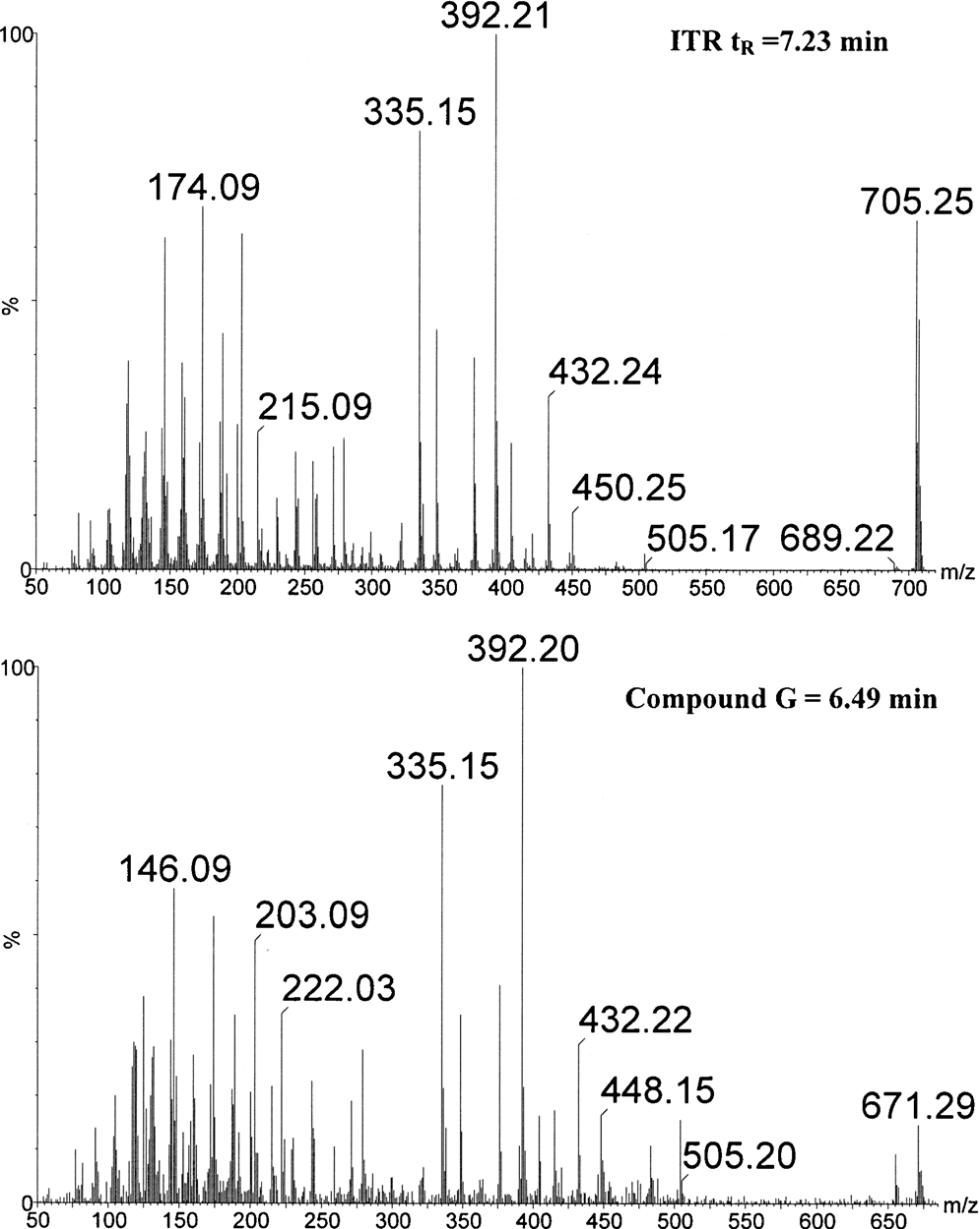
CID-MS spectra of ITR and compound G (major radiodegradation product)

Analysis of the collision-induced mass spectrometry (CID-MS) spectra recorded for the protonated ions of compounds A to J allowed to propose the structure of these compounds (Table II). It was found that the major radiodegradation products (compounds G and F) are the ITR derivatives without one chlorine atom in the phenyl ring, one in the *ortho* and the second in the *para* position (Fig. 7). Other products are ITR derivatives resulting from the following reactions:

**Table II:**
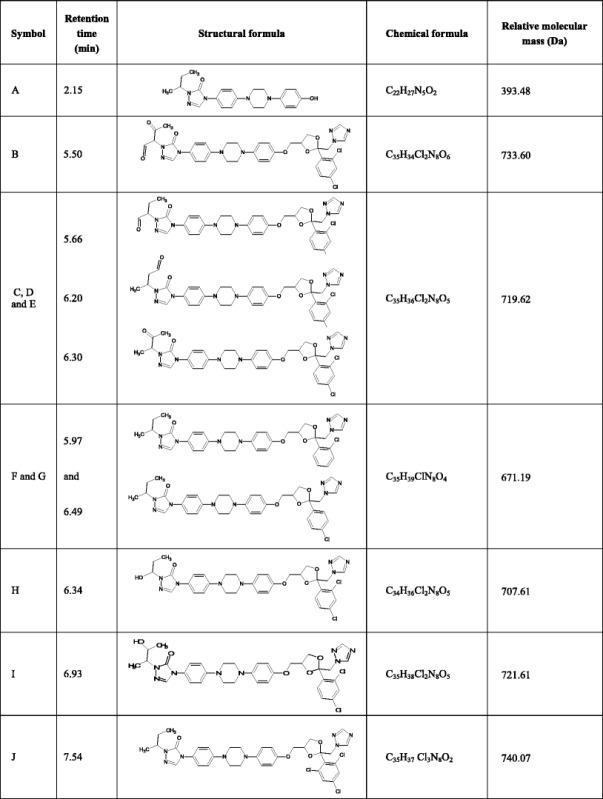
Proposed Impurities and Radiolysis Products of ITR

- Oxidation with dehydrogenation—compounds C, D and E (three isomers of the C = O group position) and compound B.
- Oxidation with methyl group removal—compound H.
- Substitution of OH groups—compound I.
- C-O bond rupture reaction—compound A.
- Trace amounts of 2,4,5-trichlorophenyl derivative (compound J) were also found in the ITR irradiated by a dose of 400 kGy which may be a by-product of the formation of dechloroitraconazole.

**Fig. 8:**
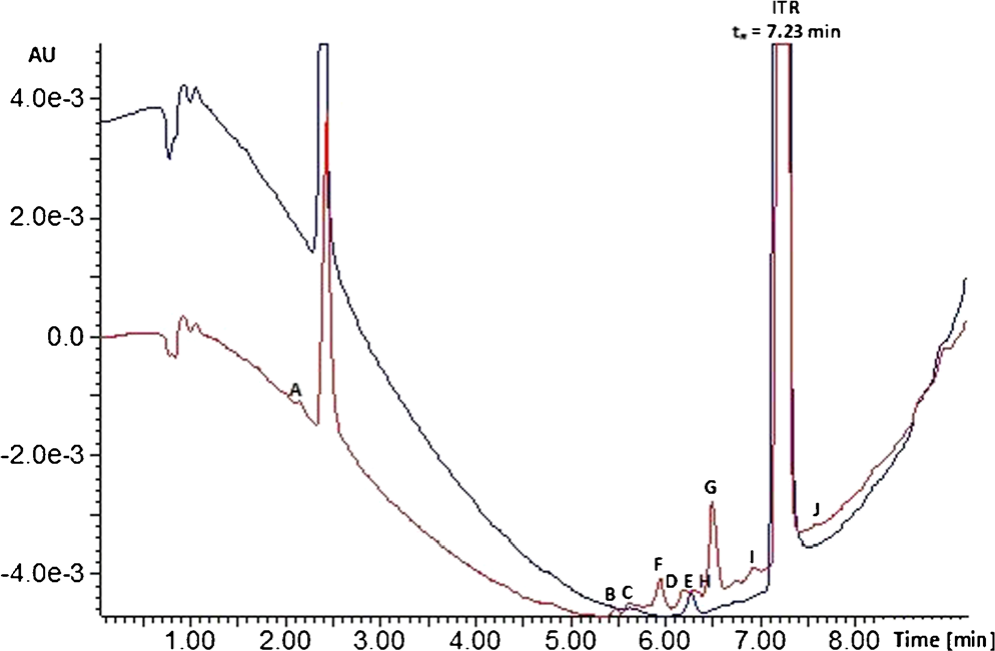
UPLC chromatograms of ITR before (*blue*) and after irradiation with 400 kGy dose (*red*)

Impurities found in the samples not exposed to irradiation are compounds formed by the oxidation mechanism. One can hypothesize that they are degradation products. Degradation proceeds very slowly at room temperature; however, under an atmosphere of air and ionizing radiation in the form of e-beam, it accelerates. Figure 8 shows the UPLC-MS chromatograms of non-irradiated (blue line) and irradiated with maximum 400 kGy dose (red line) ITR samples. The analysis revealed low levels of compounds H and E in the non-irradiated ITR samples. They could be by-products of the compound synthesis or degradation products related to the sample ageing at room temperature. The irradiation decreased the content of the ITR about 0.4% after exposure to the dose of 25 kGy and 6.5% after the dose of 400 kGy. In addition to compounds E and H, six radiodegradation products in the samples irradiated with a dose of 25 kGy were found and their number increased to eight for the dose of 400 kGy (Fig. 8, Table II).

**Fig. 9:**
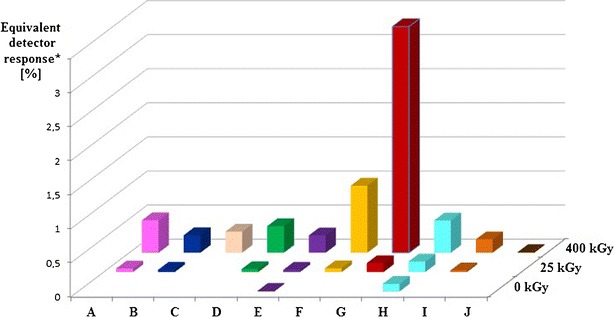
The impurity profile of ITR before (0 kGy) and after irradiation (25 and 400 kGy). *Asterisk* indicates 100% = sum of peak areas on chromatogram

The level of response of the mass detector for protonated ions of the studied compounds was used for the semiquantitative estimation of their content. Figure 9 shows the normalized recovery values. As it should be expected, these values increase with increasing doses of radiation. According to these data, compound G is the main radiodegradation product and another one is compound F. It has to be noted that the contents of compounds E and H present in the non-irradiated ITR also increased after irradiation.

**Fig. 10:**
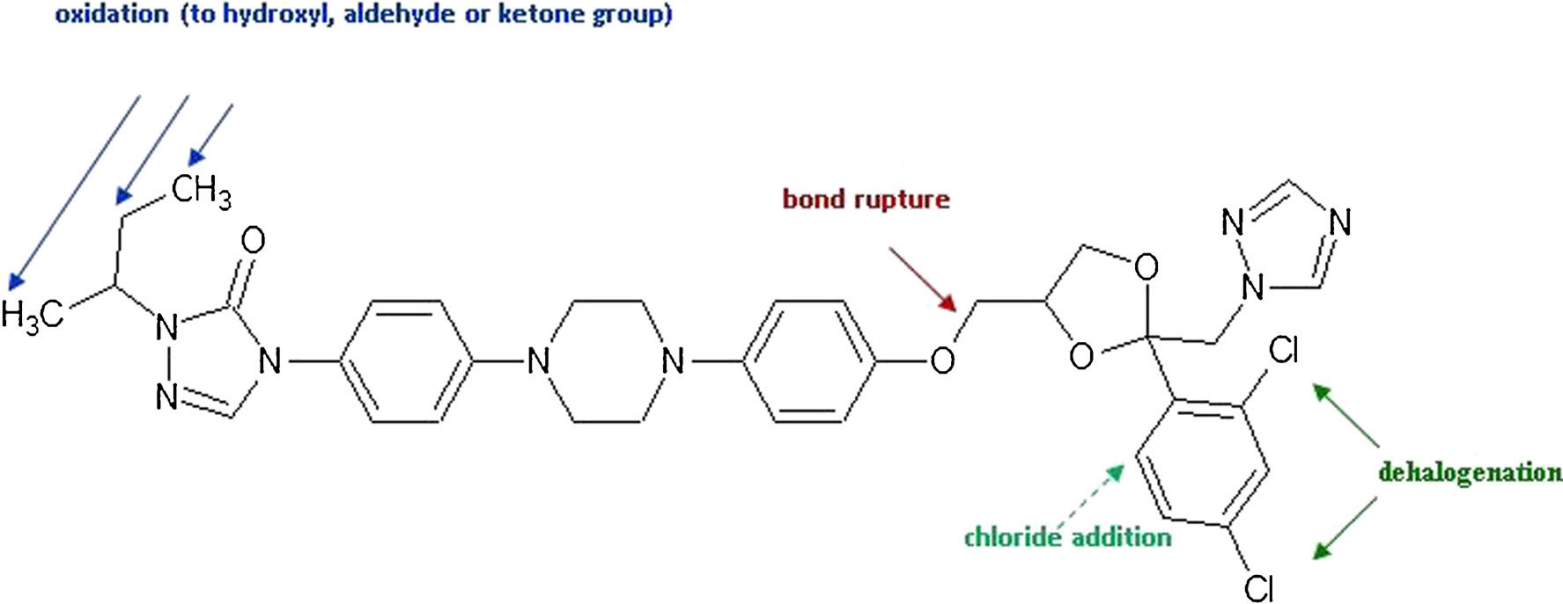
Proposed radiolysis-induced processes pathway of ITR

Main directions of radiodegradation pathway are shown in Fig. 10. It was found that monodehalogenation and oxidation reactions or C-O bonds rupture were also observed in the ketoconazole (KK) (13). These compounds have many similarities in their structures; however, they differ in the type of azole (imidazole in KK, triazoles in ITR) and the substitution of the piperazine groups (acetyl group in KK and substituent alkyltriazolone in ITR). The main radiodegradation product of KK, similarly to ITR, was a dechloro derivative and the rupture of the C-O bond occurred as well. Under the influence of e-beam in air atmosphere, ITR was oxidized within alkyltriazolone substituent while KK, without such a substituent, formed *N*-oxide within the imidazole ring. A demethylation occurred in both cases; however, due to the differences in structures, it took place in different parts of the molecules.

## CONCLUSIONS

ITR is a compound with high radiochemical stability. The standard dose (25 kGy) of radiation sterilization does not alter its physical and chemical properties that meet (capillary method—Table I) the requirements of the European Pharmacopeia 6.0 (5). It can be sterilized by radiation.

The analysis of crystalline form (X-ray diffractometry (XRD) and SEM) and spectroscopic methods (FT-IR, ultraviolet-visible spectrophotometry (UV-VIS)) did not detect changes in the properties occurring after ITR irradiation. However, DSC method has proved to be a useful tool to assess the compound degradation; a shift of the melting peak towards the lower temperatures illustrates the decrease in the ITR content.

To our knowledge, this is the first study testing the influence of ionizing radiation on itraconazole. Ten ITR derivative structures were separated by UPLC and analyzed by the CID spectra. On this basis, the main reactions of ITR radiodegradation are as follows: dehalogenation (Cl), more gradual oxidation and C-O bond rupture.
